# Model-driven intracellular redox status modulation for increasing isobutanol production in *Escherichia coli*

**DOI:** 10.1186/s13068-015-0291-2

**Published:** 2015-08-01

**Authors:** Jiao Liu, Haishan Qi, Cheng Wang, Jianping Wen

**Affiliations:** Key Laboratory of Systems Bioengineering (Ministry of Education), Tianjin University, Tianjin, 300072 People’s Republic of China; SynBio Research Platform, Collaborative Innovation Center of Chemical Science and Engineering (Tianjin), School of Chemical Engineering and Technology, Tianjin University, Tianjin, 300072 People’s Republic of China

**Keywords:** Isobutanol, Redox balance, Genome-scale metabolic model, Synthetic promoters, Glyceraldehyde-3-phosphate dehydrogenase

## Abstract

**Background:**

Few strains have been found to produce isobutanol naturally. For building a high performance isobutanol-producing strain, rebalancing redox status of the cell was very crucial through systematic investigation of redox cofactors metabolism. Then, the metabolic model provided a powerful tool for the rational modulation of the redox status.

**Results:**

Firstly, a starting isobutanol-producing *E. coli* strain LA02 was engineered with only 2.7 g/L isobutanol produced. Then, the genome-scale metabolic modeling was specially carried out for the redox cofactor metabolism of the strain LA02 by combining flux balance analysis and minimization of metabolic adjustment, and the GAPD reaction catalyzed by the glyceraldehyde-3-phosphate dehydrogenase was predicted as the key target for redox status improvement. Under guidance of the metabolic model prediction, a *gapN*-encoding NADP^+^ dependent glyceraldehyde-3-phosphate dehydrogenase pathway was constructed and then fine-tuned using five constitutive promoters. The best strain LA09 was obtained with the strongest promoter BBa_J23100. The NADPH/NADP + ratios of strain LA09 reached 0.67 at exponential phase and 0.64 at stationary phase. The redox modulations resulted in the decrease production of ethanol and lactate by 17.5 and 51.7% to 1.32 and 6.08 g/L, respectively. Therefore, the isobutanol titer was increased by 221% to 8.68 g/L.

**Conclusions:**

This research has achieved rational redox status improvement of isobutanol-producing strain under guidance of the prediction and modeling of the genome-scale metabolic model of isobutanol-producing *E. coli* strain with the aid of synthetic promoters. Therefore, the production of isobutanol was dramatically increased by 2.21-fold from 2.7 to 8.68 g/L. Moreover, the developed model-driven method special for redox cofactor metabolism was of very helpful to the redox status modulation of other bio-products.

**Electronic supplementary material:**

The online version of this article (doi:10.1186/s13068-015-0291-2) contains supplementary material, which is available to authorized users.

## Background

As an important platform chemical compound, isobutanol has been widely used in the fields of food, pharmaceutical and chemical and so on [1]. Recently, bio-produced isobutanol was considered as an ideal gasoline additive or substitute that had higher energy density and octane number, and lower hygroscopicity [2]. Thus, the isobutanol biosynthesis has attracted much attention, and several isobutanol-producing strains have been engineered by constructing the heterologous *Ehrlich* pathway in different host strains such as* Escherichia coli* [2], *Bacillus subtilis* [3], *Corynebacterium glutamicum* [4] and so on.

To efficiently improve the strains for higher yield, there has been a growing demand for rational methods, in which the computer tools based on metabolic model have played key roles [5]. In previous reports, elementary mode analysis has been applied to design isobutanol-producing strains, and the results demonstrated that in *E. coli* the balance of NADH metabolism was a crucial factor to enable the anaerobic isobutanol production [6, 7], while in *B. subtilis* the pentose phosphate (PP) pathway and transhydrogenase were predicted as important targets for higher isobutanol yield by increasing NADPH supply [3, 8]. However, to avoid a combinatorial explosion in the elementary mode analysis, metabolic models should be reduced to smaller ones, which, for example, consisted of 79 reactions for *E. coli* [6] or 131 reactions for *B. subtilis* [3]. Then, the targets for redox balance were locally predicted based on the reduced models without covering all the redox reactions, while the truth is that nicotinamide adenine dinucleotide (NAD) participated in over 300 redox reactions [9] and NADPH participated in over 100 redox reactions [10]. Therefore, it is necessary to make a global and precise prediction based on fully understanding the redox cofactors metabolism using genome-scale metabolic model (GSMM).

For a systematical targets prediction of improving redox status, all of the redox reaction engineering methods, including cofactor-swap, knockout, and overexpression, should be simulated comprehensively [11–13]. But, up to now, the redox modulation targets were usually predicted by simulating the redox reaction cofactor-swap [11], knockout [14, 15], or overexpression [16] alone, rather than together. Recently, a new developed strategy combining flux balance analysis (FBA) and minimization of metabolic adjustment (MOMA) [12] would allow the comprehensive prediction for redox modulation in isobutanol production by simultaneously simulating the three redox reaction engineering methods. Additionally, more rational potential targets would be obtained by this modeling method, while only the transhydrogenase encoded by *pntAB* was usually straightforwardly overexpressed in the traditional metabolic engineering for redox improvement [13, 17]. Furthermore, it was seemingly impossible to reach the best redox status only by direct knockout, overexpression or replacement of the target redox reaction, which may even lead to new burden on the metabolism due to redox overregulation [18, 19]. Thus, in order to get an optimal redox status, a fine-tuning of target redox reaction could be tried with the aid of gene regulatory elements. Especially, as a typical method for fine-tuning gene expression, the synthetic promoter libraries provided a powerful tool for constructing an efficient redox modulation pathway [20, 21].

In brief, this study provided a rational method to rebalance redox status for isobutanol production. The GSMM was applied to investigate the NADH and NADPH metabolism and predict the key target of redox status modulation to be glyceraldehyde-3-phosphate dehydrogenase (GAPDH). Then, a NADP^+^ dependent glyceraldehyde-3-phosphate dehydrogenase (GAPDN) pathway was designed and constructed using *gapN* from *Clostridium acetobutylicum* to simultaneously modulate the NADH and NADPH metabolism, and a further fine-tuning of *gapN* expression was performed to get a more suitable redox status with five different artificial constitutive promoters. Finally, the best strain LA09 was obtained and its fermentation properties were investigated to illustrate the effects of redox status modulation on the cell growth, isobutanol yield and byproducts’ metabolism.

## Results

### *E. coli* LA02 engineered for isobutanol production

The *pflB*-deficient *E. coli* LA01 was used as the host strain, because deleting the main byproduct formate pathway (encoded by *pfl*) enabled redox modulation to redirect the flux distribution between the reduced byproducts (lactate, ethanol and succinate) and isobutanol. The strain *E. coli* LA02 (Table 1) was engineered for isobutanol production by introducing an efficient *Ehrlich* pathway (consisting of *kivd* from *L. lactis* IL1403 and *yqhD* from *E. coli* MG1655) and biosynthetic 2-ketoisovalerate (KIV) precursor pathway (consisting of *alsS* from *B. subtilis* 168 and *ilvCD* from *E.coli* MG1655) into *E. coli* LA01. Then, it was confirmed using RT-PCR that the five genes (*alsS*, *ilvC*, *ilvD*, *kivd* and *yqhD*) were well expressed with the 2^−∆Ct^ values of 1.35 ± 0.11, 1.43 ± 0.09, 1.37 ± 0.13, 1.17 ± 0.07 and 1.21 ± 0.08, respectively. The isobutanol titer reached 2.70 g/L in 36 h with 36 g/L glucose as substrate in bath microaerobic fermentation. The lower isobutanol yield of strain LA02 without extracellular accumulation of key intermediate metabolite isobutanol indicated the lack of drive-force for isobutanol production.

**Table 1:** Strains and plasmids used in this study

Strain	Descriptions	Source
*E. coli* MG1655	Wild type	Lab collection
*B. subtilis* 168	Wild type	Lab collection
*C. acetobutylicum* ATCC 824	Wild type	Lab collection
*L. lactis* IL1403	Wild type	Lab collection
*E. coli* LA01	MG1655Δ*pflB*	Lab collection
*E. coli* LA02	Amp, Cm; strain LA01 bearing pACYCLA09 and pTRCLA10	This work
*E. coli* LA03	Amp, Cm; strain LA01 bearing pACYCLA09 and pTRCLA11	This work
*E. coli* LA04	Amp, Cm; strain LA01 bearing pACYCLA09 and pTRCLA12	This work
*E. coli* LA05	Amp, Cm; strain LA01 bearing pACYCLA09 and pTRCLA13	This work
*E. coli* LA06	Amp, Cm; strain LA01 bearing pACYCLA09 and pTRCLA14	This work
*E. coli* LA07	Amp, Cm; strain LA01 bearing pACYCLA09 and pTRCLA15	This work
*E. coli* LA08	Amp, Cm; strain LA01 bearing pACYCLA09 and pTRCLA16	This work
*E. coli* LA09	Amp, Cm; strain LA01 bearing pACYCLA09 and pTRCLA17	This work
Plasmid		
pTRC99a	*E.coli* expression vector; Amp	Lab collection
pUC18	*E. coli* expression vector; Amp	Lab collection
pACYC184	*E. coli* expression vector; tetracycline and Cm	Lab collection
pTRCLA	*E.coli* expression vector, pBR322 ori, Amp	This work
pACYCLA	*E.coli* expression vector, pACYC ori, Cm	This work
pACYCLA09	pACYC ori, Cm, P*trc*::*alsS*-*ilvCD*	This work
pTRCLA10	pBR322 ori, Amp, P*trc*:: *kivd*-*yqhD*	This work
pTRCLA11	pBR322 ori, Amp, P*trc*:: *kivd*-*yqhD*, P*gapA*::*gapN*	This work
pTRCLA12	pBR322 ori, Amp, P*trc*:: *kivd*-*yqhD*, PgapA::*gapC*	This work
pTRCLA13	pBR322 ori, Amp, P*trc*:: *kivd*-*yqhD*, BBa_J23105::*gapN*	This work
pTRCLA14	pBR322 ori, Amp, P*trc*:: *kivd*-*yqhD*, BBa_J23106::*gapN*	This work
pTRCLA15	pBR322 ori, Amp, P*trc*:: *kivd*-*yqhD*, BBa_J23118::*gapN*	This work
pTRCLA16	pBR322 ori, Amp, P*trc*:: *kivd*-*yqhD*, BBa_J23102::*gapN*	This work
pTRCLA17	pBR322 ori, Amp, P*trc*:: *kivd*-*yqhD*, BBa_J23100::*gapN*	This work

As a major drive-force for isobutanol production, intracellular NADPH was usually insufficient in *E. coli*, which had been considered as a major bottleneck for increasing isobutanol production [13, 17]. On the one hand, two molecules of NADPH were required for one molecule of isobutanol synthesized from pyruvate, but inadequate NADPH was generated mainly via transhydrogenase encoded by *pntAB* under microaerobic or anaerobic conditions [13]. On the other hand, redundant NADH accumulated via the Embden–Meyerhof–Parnas (EMP) pathway with glucose as substrate, and was reoxidized for NAD^+^ regeneration with 12.60 g/L lactate and 1.60 g/L ethanol production, which decreased the availability of pyruvate as a direct precursor of isobutanol. Consequently, isobutanol could not be efficiently synthesized, and rebalancing redox status should be performed in strain improvement. Moreover, cofactors NADH and NADPH were widely involved in over 300 redox reactions of cell metabolism [9, 22]. Especially, NADPH was known to participate in over 100 redox reactions [10]. Therefore, it was necessary to establish a rational method to efficiently regulate the redox status. In this work, GSMM was used as a powerful tool to systematically simulate the effects of cofactor engineering on the isobutanol synthesis and cell metabolism, and then predict the key target for rebalancing redox status.

### In silico potential targets identification for improving redox status

The GSMM of strain LA02 was obtained and details are presented in the “Methods” section. The metabolism of NADH and NADPH in the LA02 metabolic network was simulated by FBA algorithm and the results are shown in the Additional file 1: Figure S1. The total flux of NADH (16.20 mmol/g/h), was 4.7-fold of the total flux of NADPH, 70.7% of NADH was recycled through ethanol and lactate production, and 83.6% of NADPH was generated by accepting electron from 17.8% of NDAH via pntAB transhydrogenase. Then, 23 candidate redox targets were identified as shown in the Additional file 1: Table S4, some of which were different from the 22 potential redox targets identified by parsimonious flux balance analysis (pFBA) in the Ref. [11], and the different potential targets have been labeled in the Additional file 1: Table S4. To regulate the metabolism of redox cofactors, cofactor swapping of the reactions that generated NADH or consumed NADPH, overexpression of the reactions that generated NADH, and knockout of the reactions that consumed NADH or NADPH were respectively simulated for all the candidate reactions according to the analysis shown in Additional file 1: Figure S1. The target was finally identified with the aid of *f*_PH_, which weighted both cell growth and isobutanol production rate, and the candidate redox reaction with the highest value of *f*_PH_ was indicated as the key target [23, 24].

**Fig. 1:**
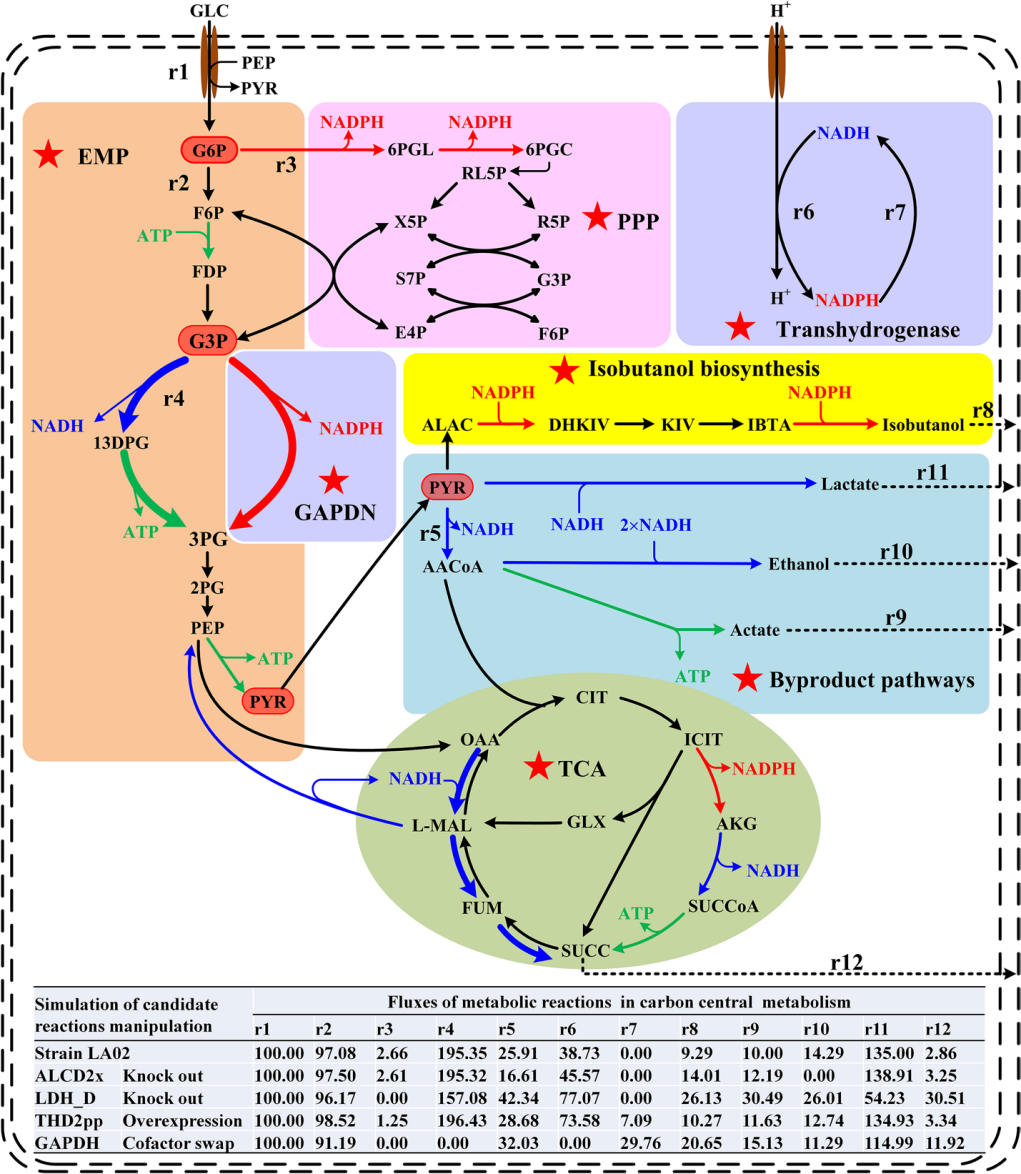
Simulation of metabolic flux changes in the central metabolism of the redox reaction manipulated strains. In each case, all fluxes were expressed in molar percentage of the corresponding glucose uptake rate. The *red lines* indicated reactions with NADP(H) as cofactor, the *thin blue lines* indicated reactions with NAD(H) as cofactor, the *green lines* indicated ATP generating/consuming reactions, the *thick blue lines* indicated the redox synthesis pathway of succinate, the *dot lines* indicated the transportation reactions from intracellular to extracellular. *1,3DPG* 3-phospho-glyceroyl phosphate, *2PG* glycerate 2-phosphate, *3PG* 3-phospho-glycerate, *6PGC* 6-phospho-gluconate, *6PGL* 6-phospho-glucono-1,5-lactone, *ACCOA* acetyl-CoA, *ALAC* 2-acetolactate, *AKG* 2-oxoglutarate, *CIT* citrate, *DHKIV* 2,3-dihydroxy-3-methylbutanoate, *E4P* erythrose 4-phosphate, *F6P* fructose 6-phosphate, *FDP* fructose-1,6-diphosphate, *FUM* fumarate, *G3P* glyceraldehyde 3-phosphate, *G6P* glucose 6-phosphate, *GLC* glucose, *IBTA* isobutaldehyde, *ICIT* isocitrate, *KIV* α-ketoisovaleric acid, *l* *-MAL* l-malate, *OAA* oxaloacetate, *PEP* phosphoenolpyruvate, *PYR* pyruvate, *R5P* ribose 5-phosphate, *RL5P* ribulose 5-phosphate, *S7P* sedoheptulose 7-phosphate, *SUCCOA* succiny-CoA, *X5P* xylulose 5-phosphate.

In the simulation, the specific isobutanol production rate could be increased only by ten potential targets manipulation (Table 2), while the modeling results of the other potential targets are presented in the Additional file 1: Table S6. With the highest value of *f*_PH_ (1.944), the reaction GAPDH was identified as the most important target. Similarly, the GAPDH was also predicted by the reference [11] to be the best cofactor-swap target in *E. coli* for enhancing the theoretical yields of several native and non-native products. The cofactor-swap of GAPDH could not only regulate the cofactor metabolism but also modulate itself carbon flux to impact the cell growth and isobutanol production. On the one hand, the limited NADPH and excess NADH were generated in *E. coli* MG1655 [25–28], which could be overcome with the new efficient NADPH-generating reaction obtained by cofactor-swap of GAPDH. On the other hand, the reaction GAPDH was a key step of the EMP pathway, which dominated the glucose catabolism, and its manipulation could increase the availability of pyruvate as the end product of the EMP pathway. The predicted specific isobutanol production was increased by 183.0%, while the specific production rate of the byproducts, ethanol and lactate, was decreased by 20.1 and 14.8%, respectively (Table 2; Fig. 1).

**Table 2:** Targets predicting for regulating redox balance to increasing isobutanol production in strain LA02

Reaction name	Reaction stoichiometry	Strategy	Special growth rate (h^−1^)	ISB special product rate (mM/g/h)	*f*_PH_
GAPDH	glyceraldehyde 3-phosphate + NAD^+^ + phosphate ↔ 3-Phospho-glyceroyl phosphate + H^+^ + NADH	Cofactor swap	0.0231	1.842	1.944
ALCD2x^a^	acetaldehyde + H^+^ + NADH → ethanol + NAD^+^	Knock out	0.0798	0.968	1.860
ACALD^a^	acetyl-CoA + H^+^ + NADH → acetaldehyde + coenzyme A + NAD^+^	Knock out	0.0876	0.717	1.118
THD2pp	NADH + NADP^+^ + H^+^(extracellular) → H^+^(intracellular) + NAD^+^ + NADPH	Over express	0.0797	0.732	1.064
GLUDy^b^	2-oxoglutarate + H^+^ + NADPH + NH_4_ ↔ L-glutamate + H_2_O + NADP^+^	Cofactor swap/knock out	0.090/0.084	0.678/0.672	1.028/0.944
FLDR2	2 flxso + NADPH → 2 flxr + H^+^ + NADP^+^	Cofactor swap	0.0925	0.671	1.035
PGCD	3-phospho-glycerate + NAD^+^ → 3-Phosphohydroxypyruvate + H^+^ + NADH	Cofactor swap	0.095	0.651	1.001
G5SD	l-Glutamate 5-phosphate + H^+^ + NADPH → L-Glutamate 5-semialdehyde + NADP^+^ + phosphate	Knock out	0.095015	0.650	0.998
G6PDH2r	glucose 6-phosphate + NADP^+^ ↔ 6-phospho-glucono-1,5-lactone + H^+^ + NADPH	Over express	0.0941	0.651	0.990
LDH_D^c^	H^+^ + NADH + pyruvate → lactate + NAD^+^	Knock out	0.005^c^	1.829	0.416

The growth and isobutanol production rates of strain LA02 were respectively 0.095 h^−1^ and 0.65 mM/g/h.

*Flxso* flavodoxin semi oxidized, *flxr* flavodoxin reduced, *ISB* isobutanol.

^a^The reactions ALCD2x and ACALD were two steps of ethanol production by the same enzyme alcohol dehydrogenase encoded by *adhE* gene.

^b^The reaction GLUDy, catalyzed by glutamate dehydrogenase consuming NADPH, could be knocked out or cofactor swapped for improving NADPH supply.

^c^The strain could not growth after LDH_D knockout with special growth rate predicted as 0.005 h^−1^.

As the next best targets, the knockouts of ethanol synthesis reactions (ALCD2x and ACALD) were obviously different from GAPDH cofactor swapping. In fact, ethanol, lactate and succinate played a similar role as the reduced byproduct which not only consumed the NADH but also reduced the carbon availability for the isobutanol production. An earlier report [29] showed that single deletion of *ldhA* (for lactate) or *adhE* (for ethanol), respectively, increased the intracellular NADH/NAD ratio by 6.4- or 0.3-fold compared to the wild-type *E. coli* strain, which provided experimental data support for the 100.2 or 18.4% increase of transhydrogenase reaction flux (THD2pp), respectively, in the knockout simulation of LDH_D and ALCD2x (Fig. 1). As a result of significant oversupply of NADH, LDH_D mutant especially stopped growing with the special growth rate (*μ*) below 0.005 h^−1^, while the *μ* of ALCD2x mutant decreased by 23.5% in the modeling; these were also observed in the experiment results of the references [29, 30]. It also has been reported that the single deletion of succinate synthesis in *E. coli* has little impact on cell growth and glucose uptake [31].

The third target transhydrogenase reaction (THD2 pp) was single overexpressed in the simulation, and the special isobutanol product rate was increased by 12.6% to 0.732 mM/g/h with the *f*_PH_ only of 1.025. Shi et al. [13] has overexpressed *pntAB* in chromosome with the expression level increased by 450-fold, but only a 21.0% increase of the isobutanol titer was observed, which was consistent with the modeling results. In fact, the manipulation of THD2pp as well as the byproduct-related reactions (ALCD2x and LDH_D) enhanced the transfer of reducing equivalents from NADH to NADPH (Fig. 1) with the energy from proton translocation as drive-force. The generation of NADPH via transhydrogenase was limited, while the required consuming energy was very expensive, especially, in micro or anaerobic condition [11]. Thus, producing NADPH directly by cofactor-swapped GAPDH was inherently more efficient than enhancing the transhydrogenase reaction [11]. Moreover, GAPDH cofactor swap allowed saving much energy by decreasing the flux of THD2 pp (the flux ratio was decreased from 38.7% to 0) (Fig. 1).

Additionally, reaction G6PDH2r catalyzed by glucose-6-phosphate dehydrogenase (encoded by *zwf* gene) had been amplified to enhance the flux of PP pathway for NADPH supply [8], but it may down-regulate the flux of EMP pathway to decrease the pyruvate availability for isobutanol production. Moreover, the *zwf* overexpression was usually coupled with the knockout of *pgi* gene in *E. coli* as reported by Ref. [32], which resulted in excess NADPH supply to inhibit cell growth with the special growth rate decreased by 78.3%. A similar result was obtained by simulation in this study, the G6PDH2r amplification was predicted to result in a slight increase of isobutanol production (0.1%) with low *f*_PH_ of 0.990 (Table 2).

Overall, the GAPDH reaction was identified to be the key target. However, it was also predicted that cofactor-swap of GAPDH resulted in a dramatically 75.0% decrease of the special growth rate, which was very consistent with experimental result of the replacement of *E. coli gapA* with NADP^+^-dependent GAPDH in the reference [28]. Thus, a GAPDN pathway was designed to regulate the cofactor metabolism for enhancing isobutanol production (Fig. 1). In this work, the GAPDN was constructed in the isobutanol-producing strain, and modulated with constitutive promoters to obtain an optimal strain.

### Model-driven rational engineering of GAPDN pathway

To construct the GAPDN pathway, heterologous NADP^+^ dependent GAPDH was applied, according to references [19, 28, 33, 34]. Especially, *gapC* from *C. acetobutylicum* had been applied to increase the NADPH pool of *E. coli* BL21 in a previous report [33]. Moreover, another isoenzyme encoded by *gapN* was also found in the same strain [34]. Isoenzymes encoded by different genes usually have different activities [2, 35], and even for a same enzyme, its activity may be greatly impacted at different intercellular environment of host strains [36]. Thus, the strains LA03 and LA04 were respectively engineered by overexpressing *gapC* and *gapN*, of which the gene expression levels were respectively, 0.271 ± 0.03 and 0.348 ± 0.04 (2^−∆*Ct*^ value). Then, the GAPDNs encoded by *gapC* and *gapN* were compared to obtain a more suitable GAPDN pathway for efficient improvement of redox status in *E. coli* isobutanol-producing strains.

**Fig. 2:**
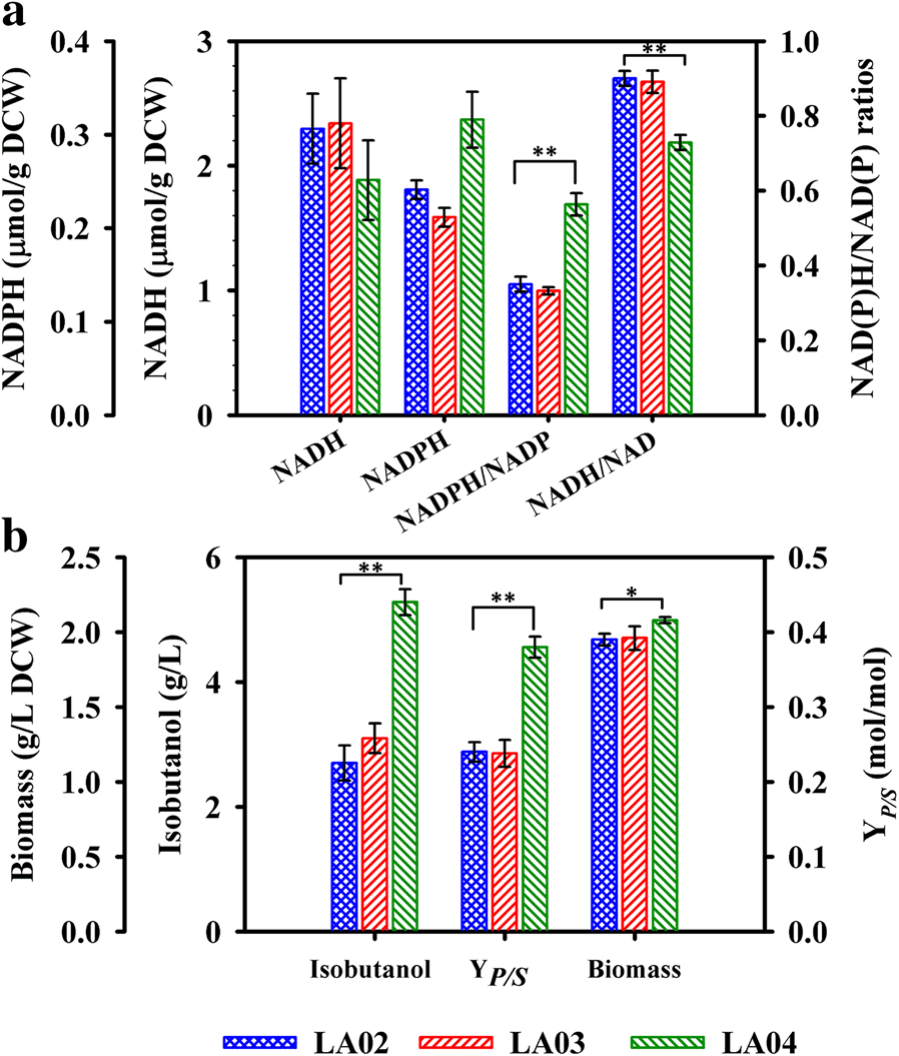
Comparison of intracellular redox cofactors in exponential phase (**a**), isobutanol and biomass (**b**) for strain LA02, LA03 and LA04. *Asterisks* indicate significant differences (*P < 0.05, **P < 0.01).

The two genes *gapC* and *gapN* were cloned and expressed in strain LA02, respectively, resulting in strains LA03 and LA04 (see Table 1). Then, the intercellular redox cofactors pool at exponential phase (EP) (18 h) and stationary phase (SP) (36 h) of bath culture were determined. Since similar results and conclusions were drawn from the data of EP and SP, the results at EP are presented in Fig. 2a and the results at SP are given in the Additional file 1: Figure S2. Previous reports had shown that the NADH/NAD^+^ ratio of the wild-type strain *E. coli* MG1655 ranged from 0.375 to 0.65, while the NADPH/NADP^+^ ratio varied from 0.65 to 0.99 [27, 28, 37]. In this work, the isobutanol was produced by the strain LA02 with lots of NADPH consumed, thus the NADPH/NADP^+^ ratio of the strain LA02 was decreased to 0.35 (EP) lower than that of the wild-type strain MG1655, while the NADH was oversupplied with a high NADH/NAD^+^ ratio of 0.90 (EP). Obviously, the redox status of strain LA02 was imbalance. As shown in Fig. 2, the redox status of strain LA03 was very close to that of strain LA02, which indicated that little improvement of the redox status had been made by *gapC* expression. Consequently, the isobutanol produced by strain LA03 was only 3.10 g/L, a slight increase compared to that of LA02 (2.70 g/L), and the isobutanol yield of LA03 (0.238 mol/mol, 28.3% of theoretical yield) was very similar to that of LA02 (0.240 mol/mol, 28.6% of theoretical yield). Unlike *gapC* expression, the *gapN* expression obviously made an increase of NADPH/NAPD^+^ ratio to 0.56 (EP) in strain LA04, 1.6-fold higher than that of strain LA02 (*P* < 0.01). Moreover, the GAPDN expression could reduce the NADH generation via the native GAPDH by competing for the same substrate of glyceraldehyde 3-phosphate (G3P). Thus, in strain LA04, the NADH/NAD^+^ ratio was decreased by 18.9% (EP) compared to that in strain LA02 (*P* < 0.01) (Fig. 2a). These results indicated the redox status in strain LA04 was improved by the *gapN* expression, and therefore the isobutanol production (5.28 g/L) and yield (0.38 mol/mol, 45.2% of theoretical yield) were both increased by 95.6 and 58.3% compared to those of strain LA02 (*P* < 0.01). Additionally, the final biomass of LA04 was also increased to 2.08 g/L dry cell weight (DCW), 6.7% higher than that of LA02 (*P* < 0.05) (Fig. 2c). No ATP release by the non-phosphorylating reaction catalyzed by GapN has been taken into account. The less ATP production would result in slow growth [38] and higher glucose uptake rate [39]. However, NADPH also played an important role in the biosynthesis of fatty acid, protein and nucleic acid, and was also a key metabolite to resist oxidative stress [40]. Rebalancing redox status with an increase of NADPH level would help the cell to resist the oxidative stress caused by isobutanol [8, 41, 42].

Moreover, the enzyme activity of cell extract was also assayed. As shown in the Additional file 1: Table S5, the strain LA03 exhibited much higher NAD^+^ dependent GAPDH activity (0.785 ± 0.05 IU/mg) rather than NADP^+^ dependent activity (0.096 ± 0.02 IU/mg), while the strain LA04 showed both of NAD^+^ and NADP^+^ dependent GAPDH activity (0.608 ± 0.03 and 0.425 ± 0.05 IU/mg, respectively). The result was well consistent with the result of previous research that GAPDN encoded by *gapN* showed an absolute specificity for NADP^+^ [34]. The GAPDH encoded by *gapC* preferred NAD^+^ rather than NADP^+^, which was consistent with the reported NAD^+^ dependent GAPDH by the Ref. [43]. Therefore, the GAPDN encoded by *gapN* had higher efficiency and was more suitable than that encoded by *gapC* for improving redox status balance of the isobutanol-producing strain. However, the slight lower NADPH/NADP+ ratio of strain LA04 compared to the wild-type redox status indicated that a further improvement could be made to obtain an optimal redox status for isobutanol production. Thus, the fine-tuning of redox status progressed in this work.

**Fig. 3:**
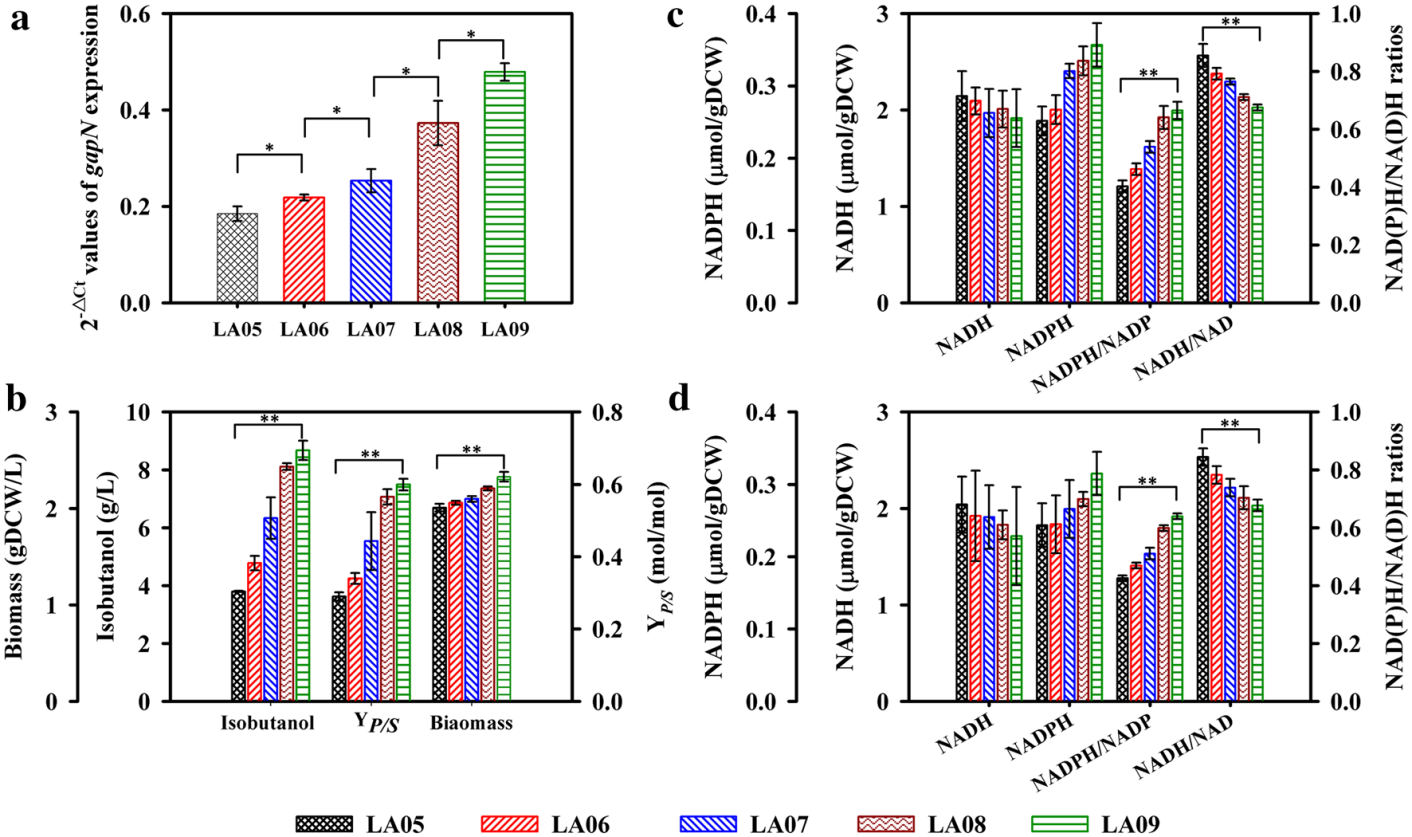
Comparison of the *gapN* expression (**a**), isobutanol and biomass (**b**), intracellular redox cofactors in exponential (**c**) and stationary (**d**) phase, for strain LA05, LA06, LA07, LA08 and LA09. *Asterisks* indicate significant differences (*P < 0.05, **P < 0.01).

Although it was impossible to define what the best redox status for isobutanol production was, a fine-tuning of *gapN* over-expression allowed to modulate NADPH and NADH generation by redistributing the fluxes between GAPDN and the native GAPDH competing for the same substrate of G3P [44]. Thus, the *gapN* gene expression was regulated under five different strength constitutive promoters in this work. These promoters BBa_J23105, BBa_J23106, BBa_J23118, BBa_J23102 and BBa_J23100 were selected from Registry of Standard Biological Parts, the promoters are listed in the order of the weakest to the strongest. The 2^−∆∆*Ct*^ values [45] of the *gapN* expression in the five strain LA05, LA06, LA07, LA08 and LA09 measured by quantitative real-time reverse transcription PCR (RT-PCR) were 0.184, 0.218, 0.253, 0.373 and 0.479, respectively (Fig. 3a), indicating a fine-tuned expression of *gapN* was achieved and would be helpful for get a moderate redox status.

Although the NADH generated by GAPDH and NADPH generated by GAPDN were hardly to be quantified [13], the correlations between *gapN* expression level and the redox status were measured by determining the total intercellular redox cofactors pool. The results showed that as the *gapN* expression level grew up, the intercellular NADPH pool increased from 0.25 to 0.36 μmol/g DCW at EP and from 0.24 to 0.32 μmol/g DCW at SP, the corresponding NADPH/NADP^+^ ratios also respectively shifted from 0.40 to 0.67 at EP (*P*<0.01) and from 0.43 to 0.64 at SP (*P* < 0.01) (Fig. 3c, d). More NADPH was generated with the flux of GAPDN increased, which would result in a decrease flux of GAPDH to down-regulate NADH production. Hence, the NADH/NAD+ ratios were decreased from 0.86 to 0.68 at EP (*P* < 0.01) and from 0.84 to 0.67 at SP (*P* < 0.01) (Fig. 3c, d). As above analysis, modulate *gapN* expression by the promoters was an efficient method to improve the redox status. Moreover, as the *gapN* expression level increased, the isobutanol production and its yield were respectively improved from 3.80 to 8.68 g/L (*P* < 0.01) and from 34.5 to 71.4% of theoretical yield (*P* < 0.01), and showed a positive correlation to NADPH/NADP^+^ ratio (the Spearman coefficient of correlation reached to 1 and *P* value was lower than 0.05) (Fig. 3b). Additionally, the biomass was also enhanced from 2.01 to 2.33 g/L (*P* < 0.01) (Fig. 3b). Eventually, the strain LA09 exhibited the best performance, and the isobutanol titer and yield were, respectively, increased by 64.4 and 57.9% compared to those of the strain LA04.

Significantly, the *gapN* expression in isobutanol production strain was also simulated based on the metabolic network model. Unlike the above experimental results, an optimal point was predicted to be present when the flux of GAPDN reached to 27.1% of the total flux from G3P to 3-phosphoglycerate (3PG), beyond which to further increase the flux of GAPDN would not lead to a better performance. The optimal redox states were also verified to be present both for butanol synthesis [44] and lysine synthesis [18]. Especially, the optimal redox states could be obtained with fine-tuning the generation of NADH by NAD^+^ dependent formate dehydrogenase [44], or NADPH by engineered GAPDH [18]. In this study, the isobutanol of the strain LA09 was increased by 5.98 g/L than that of strain LA02, requiring extra NADPH (80.8 mmol/L). Thus, it was calculated that to generate the extra NADPH, approximately 41.3% of the total consumed glucose should be catabolized through the GAPDN, which was inconsistent with the prediction of model. Thus, it demonstrated that modulation of the *gapN* expression was necessary for efficiently improving redox status, which assisted to overcome the model prediction limitations. In this work, not only the isobutanol synthesis and cell growth, but also the byproducts’ synthesis and glucose consumption of the best performing strain LA09 were investigated to illustrate the effects of rebalancing redox status on cell metabolism, and provide evidences for further study.

### Bath fermentation properties of strain LA09

**Fig. 4:**
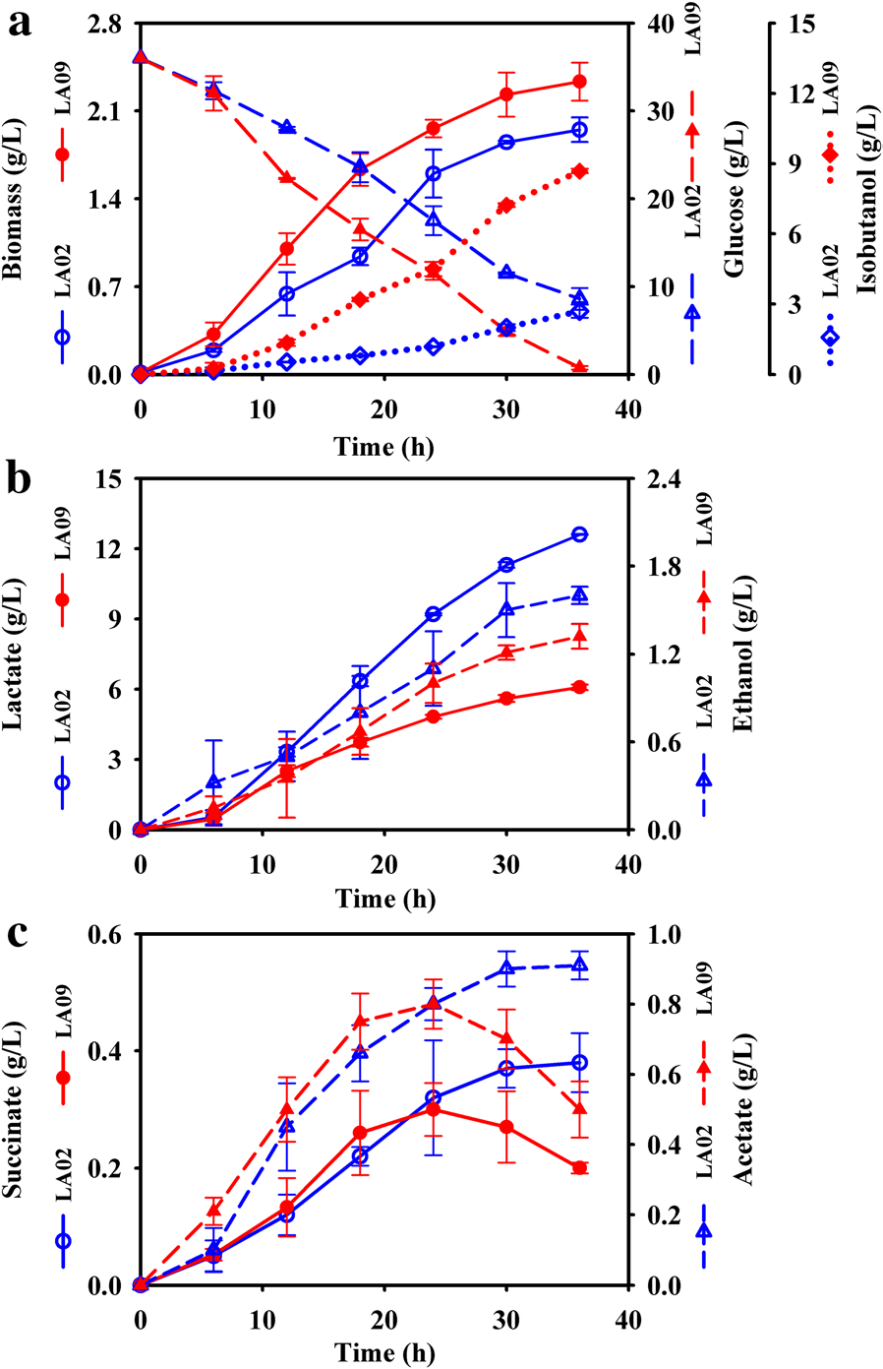
Batch fermentation properties of LA02 and LA09.

The bath fermentation properties of strain LA09 was investigated using strain LA02 as the control, and the results were given in the Fig. 4. The biomass and isobutanol in the batch fermentation of strain LA09 reached to 2.33 and 8.68 g/L within 36 h, respectively, 19.5 and 221.0% higher than that of strain LA02. The *gapN* expression not only improved the redox status of strain LA09, but also accelerated the glucose catabolism. A great majority of glucose (35.25 g/L) was consumed by strain LA09 with the special consumption rate of 0.834 g/g DCW/h, 7.65% higher than the special consumption rate of strain LA02 (0.775 g/g DCW/h). Moreover, besides isobutanol, other redox reactions of byproducts were impacted by the redox status regulation with *gapN* expression, especially, as for the down-regulated NADH pool and NADH/NAD^+^ ratio, the NADH-dependent ethanol and lactate of the stain L09 were respectively decreased to 1.32 and 6.08 g/L, 17.5 and 51.7% lower than those of strain LA02. Additionally, similar quantity of acetate and succinate were secreted by both strains. The strain LA02 finally produced 0.33 g/L succinate and 0.90 g/L acetate, but the two byproducts produced by strain LA09 reached its peaks at EP, 0.30 and 0.80 g/L, respectively, and then were re-assimilated at SP, while the glucose was nearly used up. The reuse of succinate and acetate with glucose presented by *E. coli* was also observed in previous report [46–48]. The reason may be that the secreted acetate and succinate would be re-used as carbon sources while the glucose was nearly used up. The results demonstrated that the moderate improvement of redox status based on the *gapN* expression modulation made a great and efficient strain improvement for isobutanol producing.

## Discussion

In this work, the strain *E. coli* LA02 was engineered for isobutanol production by combining NADP^+^ dependent *Ehrlich* pathway and biosynthetic KIV precursor pathway, but, as the key limitation for isobutanol production, the imbalance redox status with excess NADH and less NADPH supply should be solved to improve the strain performances. Actually, several studies had focused on the redox balance for increasing isobutanol production, including construct a isobutanol pathway using the redundant NADH as cofactor [17], or forcing high flux through the NADPH generating pathway, such as transhydrogenase [13, 17] and PP pathway [8]. Especially, Qi et al. [8] used a compacted metabolic model to predict the targets for enhance isobutanol by *B. subtilis* using the Elementary mode analysis, and a strategy of *pgi* deletion and *zwf* overexpression was carried out with the EMP pathway disrupted, and resulted in excess supply NADPH to inhibit cell growth, which was overcome by expressing the UdhA from *E. coli*. However, the GAPDH involving in the EMP pathway was rationally predicted as a new key target for redox status improvement based on the GSMM in this study. Unlike the overexpression of NADPH generating pathway, the cofactor-swap of GAPDH could not only introduce a new pathway to efficiently generate NADPH depending on the high-flux dominate EMP pathway, but also eliminate the major NADH generation pathway carrying 83.6% of total NADH generation flux according model analysis (Additional file 1: Figure S1). In practice, the cofactor-swap of GAPDH could be achieved by two approaches, one of which was to change the cofactor preference of the *E. coli* native glycerate-3-phosphate dehydrogenase by enzyme engineering, but it was seemingly difficult to be applied for fine-tuning the redox status because the engineered enzymes usually had catalytic efficiency both towards NAD^+^ and NADP^+^, rather than used NADP^+^ as the sole cofactor according to description in previous [18]. Fortunately, as another approach, heterologous GAPDN that catalyzed the irreversible reaction from G3P to 3PG had been found in some gram-positive bacteria such as *Streptococcus* and *Clostridium* species [34]. In this work, the *gapN* gene was obtained from *C. acetobutylicum* to encode the GAPDN pathway.

Furthermore, two methods should be considered for engineering the target reaction, including replacing the native GAPDH with the GAPDN or directly heterologous overexpressing the GAPDN. For the first one, it may result in overregulation of redox status with redundant NADPH generation to cause new burden on the cell metabolism [28]. Takeno et al. replaced the NAD^+^ dependent GAPDH of *C. glutamicum* strain with GAPDN from *S. mutans*, and the mutant strain showed significant growth deficiency [19]. In this case, the result was very consistent with the prediction of metabolic network modeling that the cofactor-swap of GAPDH decreased the specific growth rate of strain by 75.0% (Table 2). For the second method, it could provide a moderate improvement of redox status rather than overregulation. It has been proved feasible and practical to overexpress the *gapN* in the strain LA04, of which the intracellular NADPH/NADP^+^ ratio increased by 60.0% (EP) with the isobutanol production and yield respectively increased by 95.6 and 58.3% compared to those of the stain LA02. Centeno-Leija et al. [28] also demonstrated that overexpression of GAPDN showed a better promising performance than the replacement of native GAPDH with GAPDN in modulating NADPH supply to increase polyhydroxyalkanoates production.

However, it was seemingly unable to get an optimal redox status for maximizing the isobutanol production only by expression of GAPDN under the *gapA* promoter, and the experiment results also demonstrated the intracellular NADPH/NADP^+^ ratio in strain LA04 (0.56 at EP) was still lower than that in the wild-type (0.65–0.99). Thus, a further modulation was necessary for increasing NADPH generation to satisfy the demand of isobutanol biosynthesis. In this work, a recovery redox status to wild-type state was achieved by a step by step increase of NADPH generation based on the modulation GAPDN pathway under the regulation of five promoters with gradual-increasing strength, and a strain LA09 of the best performance was obtained with the strongest promoter BBa_J23100. It might be doubtful that if more isobutanol would be produced with a promoter stronger than BBa_J23100 placed in front of GAPDN. But *gapN* has been expressed under the Trc promoter, and the cell growth of *E. coli* strain was decreased [28]. Thus, it did not have to get more isobutanol production with a stronger promoter. Finally, the isobutanol production and yield of the best strain LA09 were respectively increased by 2.21- and 1.50-fold compared to that of strain LA02, while the productions of byproducts (ethanol and lactate) respectively decreased by 17.5 and 51.7%. Nevertheless, as branch pathways, the biosynthesis of ethanol and lactate should be completely eliminated to reroute more carbon flux into isobutanol synthesis in future work, and then, for the new strain, it would be also need to further fine-tune the fluxes of GAPDH and GAPDN pathway to get optimal redox status for high efficient isobutanol production.

Above all, as a key limitation of isobutanol production, the redox status imbalance was solved by a rational method in this work. The GSMM was used as a powerful platform to gain insight into the redox cofactors metabolism, and the most important target was predicted to be the GAPDH. Based on the model analysis and prediction, the GAPDN pathway encoded by *gapN* was consequently rationally designed, and then modulated with the help of artificial, constitutive promoters to improve the redox status. Finally, an efficient stain LA09 was obtained. Collectively, it indicated the GSMM combination with synthetic promoters was a powerful and promising strategy for the rational improvement of redox status, and this strategy could also be utilized to efficiently construct or optimize other cell factories for various chemicals and fuels production.

## Conclusions

In this work, a rational strategy combining GSMM and synthetic promoters was employed to rebalance redox status for higher isobutanol production. A strain LA02 was firstly engineered with NADPH-dependent isobutanol synthesis pathway including heterologous Ehrlich pathway (consisting of kivd and yqhD genes) and biosynthetic 2-ketoisovalerate precursor pathway (consisting of alsS, ilvC and ilvD genes). But the isobutanol production of strain LA 02, only 2.7 g/L with 36 g/L glucose as substrate, was limited by the imbalance of redox status. The metabolism of NADH and NADPH in the GSMM was deeply investigated with the combination of FBA and MOMA algorithms, and the key target for rebalancing the redox status was predicted to be GAPDH. Consequently, a GAPDN pathway was designed and implemented experimentally to simultaneously modulate the NADH and NADPH generation. Abilities of two GAPDNs encoded by gapC and gapN from Clostridium acetobutylicum to improve redox status were compared, and the NADPH/NADP + ratios in the strain LA04 (gapN expressed) were 0.56 and 0.60 at exponential phase and stationary phase, respectively, 1.697- and 1.714-fold of that in strain LA03 (gapC expressed). The higher NADPH/NADP+ ratio indicated gapN-encoding GAPDN was more efficient to modulate redox status. Hence, gapN was the better choice. Furthermore, a suitable flux through GAPDN pathway was necessary to get a best redox status for maximizing the isobutanol production, thus, a fine-tuning of gapN expression was progressed using five different artificial constitutive promoters. As the strengths of promoters increased, the NADPH/NADP + ratios were shifted from 0.40 to 0.67 at exponential phase and from 0.43 to 0.64 at stationary phase. The best strain LA09 was finally obtained with the gapN expressed under the control of BBa_J23100 promoter. The isobutanol production of strain LA09 reached to 8.68 g/L, 2.21-fold higher than that of strain LA02, while the ethanol and lactate productions were respectively decreased by 17.5 and 48.5% to 1.32 and 6.08 g/L. The present study highlights the power and promise of the rational method combining GSMM and synthetic promoters in redox status modulation.

## Methods

### Bacterial strains and plasmids

The bacterial strains and plasmids used in this study are given in Table 1, and all the primers used in this study are found in Additional file 1: Table S1. *E. coli* LA01 was used as the initial host. The gene-manipulation techniques for strains and plasmids construction followed standard protocols [49] and the detailed approaches are described in the following subsections. Unless otherwise noted, all enzymes were purchased from Fermentas Co., Ltd (Glen Burnie, MD, USA). Oligonucleotides were synthesized by Invitrogen Biotechnology Co., Ltd (Carlsbad, CA, USA), and DNA sequencing was served by BGI (Beijing, China).

### Construction of *Ehrlich* pathway and the biosynthetic KIV precursor pathway for isobutanol production

Isobutanol bio-production could be launched in *E. coli* by engineered heterogeneous *Ehrlich* pathway and the biosynthetic KIV precursor pathway [2]. For expression of the two pathways, two compatible vectors pTRCLA and pACYCLA were first constructed and details are provided in Additional file 2.

For *Ehrlich* pathway construction, the *kivd* gene was amplified from *Lactococcus lactis* IL1403 with primer set kivd-F/kivd-R and digested with *BamH*I and *Xba*I and cloned into pUC18 cut with the same enzymes, creating pUCLA01. The *yqhD* gene from *E. coli* MG1655 was amplified with primer set yqhD-F/yqhD-R and digested with *Pst*I and *Xba*I and cloned into pUCLA01 cut with the same enzymes, constructing pUCLA02. The plasmid pUCLA02 was then digested with *BamH*I and *Xba*I, and the fragment containing *kivd*-*yqhD* sequence was cloned into pTRCLA cut with the same enzymes, producing pTRCLA10.

The precursor pathway was constructed as follows: The *alsS* gene from *Bacillus subtilis* 168 was amplified with primer set alsS-F/alsS-R and digested with *BamH*I and *Sal*I and cloned into pUC18 cut with the same enzymes, resulting in pUCLA03. The *ilvC* gene from *E. coli* MG1655 amplified with primer set ilvC-F/ilvC-R was digested with *Mlu*I and *sal*I and cloned into pUCLA03 cut with the same enzymes, creating pUCLA04. The *ilvD* gene from *E. coli* MG1655 was amplified with primer set ilvD-F/ilvD-R and digested with *Bgl*II and *sal*I and cloned into pUCLA04 cut with the same enzymes, producing pUCLA05. The plasmid pUCLA05 was digested with *BamH*I and *Sal*I, and the fragment including *alsS*-*ilvCD* sequence was cloned into pACYCLA cut with the same enzymes, generating pACYCLA09.

Finally, pACYCLA09 and pTRCLA10 were both transformed into *E. coli* LA01, generating the preliminary isobutanol-producing *E. coli* strain LA02.

### GSMM reconstruction and targets prediction for redox balance to enhance isobutanol production

For systematically investigating redox cofactors (NADH and NADPH) metabolism, the latest GSMM iJO1366 of *E. coli* MG1655 [50] was used as the base model in this study. The transhydrogenase reaction encoded by *pntAB* (THD2pp) was modified according to the previous report [51], and details are listed in Additional file 1: Table S2. Then, two metabolic reactions involved in *Ehrlich* pathway from KIV to isobutanol and three isobutanol transport reactions from intracellular to extracellular were also added into the model; the added reactions are given in Additional file 1: Table S3. The final GSMM proved to be reasonable by the verification, and could be applied for the metabolic simulation of the strain* E. coli* LA02. The details of model verification were given in Additional file 3.

In order to reduce computational effort, the sets of candidate oxidoreductase reactions available for modification should be determined according to previous reports [52]. In the GSMM, all reactions, NAD(H) or NADP(H) that participated were located. Then, the reactions carrying no flux, essential reactions, orphan and spontaneous reactions were removed; the reactions involved in the subsystems (cell envelope biosynthesis, glycerophospholipid metabolism, inorganic ion transport and metabolism, ipopolysaccharide biosynthesis and recycling, membrane lipid metabolism, murine biosynthesis and recycling, tRNA charging, and inner/out membranes transports) were excluded. Finally, the remaining sets of reaction were used as candidates for target prediction of rebalancing redox status (the candidate reactions are listed in the Additional file 1: Table S4). The targets prediction was performed using the COBRA Toolbox v2.0 in MATLAB (The MathWorks, Inc., USA) version 8.1 with Gurobi version 5.6.0 (Gurobi Optimization, Inc., USA) according to Ref. [12, 23] and details are as follows.

The initial fluxes of candidate reactions were first obtained using FBA algorithm with maximization of specific growth rate as the objective function and the experimental constraint (see Additional file 3). Secondly, for the knockout simulation, the flux of candidate reaction was set to be zero; for the overexpression simulation, the flux of candidate reaction was amplified to *N*-fold of the initial flux (*N* = 1.1, 1.2, 1.3… and 2.0); for cofactor swap simulation, non-native oxidoreductase reaction, obtained from the candidate reaction by cofactor swapping, are added to the system. The flux of native candidate reaction was set to be zero and the flux of the added reaction was set to be *M*-fold of the initial flux of native reaction (*M* = 0.1, 0.2, 0.3… and 2.0). The setting values of *N* and *M* were derived from the modeling methods reported by Huang et al. [12].

Finally, the quadratic programming problem was solved by MOMA, which was performed by searching for the minimal Euclidean distance with the same metabolic model. The targets were identified through comparing the phenotypic fraction value, *f*_PH_ (the ratio of weighted and dimensionless specific growth rate and specific isobutanol production rate) [12, 23, 24].

$$f_{\text{PH}} = f_{\text{biomass}} \times f_{\text{isobutanol}}^{2} = \left( {\frac{{v_{\text{biomass, modification}} }}{{v_{\text{biomass,initial}} }}} \right) \times \left( {\frac{{v_{\text{isobutanol, modification}} }}{{v_{\text{isobutanol,initial}} }}} \right)^{2}$$

The reaction that had the higher *f*_PH_ was considered as the better candidate to rebalance redox state for enhancing isobutanol production.

### Construction and regulation of GAPDN pathway

The GAPDN pathway was designed according to the target prediction. In order to obtain an efficient GAPDN, a comparison between two GAPDNs encoded by *gapN* and *gapC* was progressed under the control of *E. coligapA* promoter (P*gapA*). Firstly, the P*gapA* was amplified from *E. coli* MG1655 with primer set PgapA-F/PgapA-R and digested with *Mlu*I and *Kpn*I and clone into pUC18, creating pUCLA06. Secondly, the *gapN* and *gapC* genes from *C. acetobutylicum* ATCC 824 were amplified with two pairs of primers gapN-F/R and gapC-F/R, respectively, and both digested with *Mlu*I and *Sal*I and cloned into pUCLA06, creating corresponding pUCLA07, and pUCLA08. Finally, pUCLA07, and pUCLA08 were both digested with *Kpn*I and *Sal*I, two fragments PgapA::gapN and PgapA::gapC were cloned into pTRCLA10, creating pTRCLA11 and pTRCLA12.

Secondly, the expression of *gapN* gene was modulated using five constitutive promoters (BBa_J23105, BBa_J23106, BBa_J23118, BBa_J23102, and BBa_J23100) from the Registry of Standard Biological Parts (http://partsregistry.org/). These five constitutive promoters were synthetized in the primers BBa_J23105-F, BBa_J23106-F, BBa_J23118-F, BBa_J23102-F, and BBa_J23100-F, respectively. A fragment containing *gapN* gene under the control of BBa_J23105 promoter was amplified from pUCLA07 using the primer set BBa_J23105-F/gapN-R and digested with *KpnI* and *SalI*, then cloned into pTRCLA10, resulting in pTRCLA13. For expressing *gapN* under the other four promoters, the plasmids pTRCLA 14–17 were also constructed in the same manner.

### Medium and culture conditions

For gene manipulation, *E. coli* were cultured in Luria–Bertani medium (10 g/L peptone, 5 g/L yeast extract, and 5 g/L NaCl) at 37°C. Isobutanol fermentation were performed using M9 medium supplemented with 36 g/L glucose, 5 g/L yeast extract, and 1,000th dilution of Trace mix A5. Ampicillin and chloramphenicol (Sangon Biotech, Shanghai, China) were added appropriately. M9 media contained 17 g/L Na_2_HPO_4_·12H_2_O, 3 g/L KH_2_PO_4_, 0.5 g/L NaCl, 1 g/L NH_4_Cl, 0.24 g/L MgSO_4_, and 0.011 g/L CaCl_2_. Trace mix A5 was composited of 2.86 g/L H_3_BO_3_, 1.81 g/L MnCl_2_·4H_2_O, 0.222 g/L ZnSO_4_·7H_2_O, 0.39 g/L NaMoO_4_·2H_2_O, 0.079 g/L CuSO_4_·5H_2_O, and 49.4 mg/L Co(NO_3_)_2_·6H_2_O [2].

Pre-cultures of isobutanol fermentation were performed at 37°C in test tubes containing 3 mL of the supplemented M9 medium for 12 h. Fermentations were inoculated with 1% pre-cultures in 250 mL flasks containing 20 mL the supplemented M9 medium. After 4 h cultivation at 37°C at 250 rpm, cells were grown to an optical density at 600 nm of 0.7; subsequently, 0.1 mM isopropyl-β-d-thiogalactoside (Sigma-Aldrich, St. Louis, MO, USA) was added. The flasks were sealed tightly with rubber stoppers. Then, microaerobic cultivation was performed at 30°C at 250 rpm for 32 h.

### Expression level of *gapN* gene detected by RT-PCR analysis

The expression levels of *gapN*, *gapC*, *alsS*, *ilvC*, *ilvD*, *kivd* and *yqhd* were determined by RT-PCR, according to references [45, 53]. The recombinant strains were cultured in M9 medium supplemented with 36 g/L glucose, 5 g/L yeast extract, and 1,000th dilution of Trace mix A5. Cells were harvested when OD_600_ reached 4.0. Total mRNA was extracted using the CellAmp^®^ Direct RNA Prep Kit (Takara, Dalian, China) as described by the manufacturer. The cDNA was amplified using PrimeScript™ RT reagent Kit (Takara, Dalian, China) with the total mRNA as the templates. Samples were then analyzed using Bio-Rad iQ5 Real Time PCR (Bio-Rad, USA) with SYBR^®^ Premix Ex Taq™ (Takara, Dalian, China). RT-PCR amplification primers are given in Additional file 1: Table S1. The 16SrRNA gene was selected as internal standard. The obtained data were analyzed by using the 2^−ΔΔ*Ct*^ method described previously [45].

### Determination of NAD(P)^+^ and NAD(P)H concentrations

The intracellular NAD(P)^+^ and NAD(P)H were determined by high-performance liquid chromatography (HPLC) following extraction as in previous descriptions [54, 55]. Cells were harvested by centrifugation (4°C, 1 min, 10,000 rpm) at exponential phase (18 h) and stationary phase (36 h); the pellet was resuspended in 1.0 mL of 0.3 M HCl to extract the oxidized forms or 1.0 mL of 0.3 M KOH to extract the reduced forms. After heating at 50°C for 10 min, all samples were cooled on ice to 0°C. And then neutralization was performed by adding 0.3 mL of 0.1 M KOH for oxidized forms or 0.3 mL of 0.1 M HCl for reduced forms. The cellular debris was removed by centrifuging (4°C, 5 min, 15,000 rpm). Supernatants were transferred to new tubes. Then the NAD(P)^+^ and NAD(P)H were determined by a HPLC system using Synergi™ Hydro-RP column (250 mm × 4.6 mm, 4 μm, Phenomenex, USA) as column with operating temperature at 30°C. The mobile phase consisted of 80% A and 20% B (A: 0.2 M phosphate buffer containing 10 mM tetrabutyl ammonium bromide, pH 7.0; B: methanol). The flow rate was 0.8 mL/min and the detection wavelength was 254 nm. The standards of cofactors were purchased from Sigma-Aldrich (St. Louis, MO, USA).

### Enzyme activity assay of GAPDH in cell-free extracts

The enzyme activity was measured as described by Iddar et al. [34] and Centeno-Leija et al. [28]. Cells were harvested by centrifugation (6,000 rpm, 10 min, 4°C), and then washed once with 20 mM Tricine buffer (containing 3 mM 2-mercapto-ethanol, pH 8.5). The resuspended cells in 500 μL of the same buffer were sonicated for five pulses of 30 s and pauses of 30 s on ice with a sonicator (Laballiance SL-600S, USA). After sonication, the resulting supernatant was obtained by centrifugation (10,000 rpm, 10 min, 4°C), and used for the enzymatic assays. The NADP^+^ dependent GAPDH reaction was started by adding the cell extract to the assay solution (containing 20 mM Tricine buffer, 3 mM 2-mercapto-ethanol, 1 mM NADP^+^, and 1 mM d-glyceraldehyde-3-phosphate, pH 8.5) at 25°C. The NAD^+^ dependent GAPDH reaction was measured at 25°C in the assay solution (containing 20 mM Tricine buffer, 3 mM 2-mercapto-ethanol, 1 mM NAD^+^, 10 mM AsO_4_^3−^, and 1 mM d-glyceraldehyde-3-phosphate, pH 8.5). The molar extinction coefficient of NADH or NADPH at 340 nm is 6,220 L mol^−1^ cm^−1^.One specific unit of activity is defined as 1 μmol of NAD(P)H formed per min per mg of protein (IU/mg). The protein concentration was determined by the Bradford assay.

### Analytical methods

Cell growth was monitored by optical density measurements at 600 nm and converted to DCW concentration using the value 0.332 g/L DCW per unit OD as determined in this study. Glucose concentration was determined with a biosensor analyzer (SBA-40C, Shandong, China). To quantify organic acids, sample (10 μL) was injected into (HPLC) (1200, Agilent Technologies, USA) equipped with Synergi™ Hydro-RP column (250 mm × 4.6 mm, 4 μm, Phenomenex, USA). The mobile phase was 5 mM H_2_SO_4_ at a flow rate of 0.8 mL/min with detection wavelength of 210 nm. The column was operated at 30°C. Isobutanol, ethanol and isobutanol were quantified by a gas chromatograph (430-GC, Bruker, USA) with a flame ionization detector. A 30 m, 0.32 mm i.d., 0.25 μm BW-SWAX capillary column was used. GC oven temperature was initially held at 80°C for 2 min, and then raised to 120°C with a gradient 10°C/min and held for 2 min. And then it was raised with a gradient 50°C/min until 230°C and held for 2 min. Nitrogen was used as the carrier gas at 1.0 mL/min. The sample of 1 μL was injected with 1-butanol as internal standard. Unless otherwise noted, high purity chemical standards were all purchased from Sangon Biotech (Shanghai, China).

### Statistical analysis

Three independent biological replicates were performed for every sample, and the experimental data were calculated as the mean value with the error indicated by the standard deviation. Student’s *t* test was used to evaluate differences between the experimental data with the SPSS software 19.0 (IBM, USA).

## Additional files

Additional file 1: Supplementary supporting data. Additional file 2: Construction procedures of pTRCLA and pACYCLA. Additional file 3: Model verification.

## Abbreviations

3PG: 3-phosphoglycerate
DCW: dry cell weight
EMP pathway: Embden–Meyerhof–Parnas pathway
EP: exponential phase
FBA: flux balance analysis
G3P: glyceraldehyde 3-phosphate
GAPDH: glyceraldehyde-3-phosphate dehydrogenase
GAPDN: NADP^+^ dependent glyceraldehyde-3-phosphate dehydrogenase
GSMM: genome-scale metabolic model
HPLC: high-performance liquid chromatography
KIV: 2-ketoisovalerate precursor
MOMA: minimization of metabolic adjustment
NAD: nicotinamide adenine dinucleotide
NADH: reduced nicotinamide adenine dinucleotide
NAD^+^: oxidized nicotinamide adenine dinucleotide
NADPH: reduced nicotinamide adenine dinucleotide phosphate
NADP^+^: oxidized nicotinamide adenine dinucleotide phosphate
PP pathway: pentose phosphate pathway
RT-PCR: quantitative real-time reverse transcription PCR
SP: stationary phase

## References

[1] Karabektas M, Hosoz M (2009). Performance and emission characteristics of a diesel engine using isobutanol-diesel fuel blends. Renew Energy.

[2] Atsumi S, Hanai T, Liao JC (2008). Non-fermentative pathways for synthesis of branched-chain higher alcohols as biofuels. Nature.

[3] Li S, Huang D, Li Y, Wen J, Jia X (2012). Rational improvement of the engineered isobutanol-producing *Bacillus subtilis* by elementary mode analysis. Microb Cell Fact.

[4] Smith KM, Cho KM, Liao JC (2010). Engineering *Corynebacterium glutamicum* for isobutanol production. Appl Microbiol Biotechnol.

[5] Ghosh A, Zhao H, Price ND (2011). Genome-scale consequences of cofactor balancing in engineered pentose utilization pathways in *Saccharomyces cerevisiae*. PLoS ONE.

[6] Trinh CT, Li J, Blanch HW, Clark DS (2011). Redesigning *Escherichia coli* metabolism for anaerobic production of isobutanol. Appl Environ Microbiol.

[7] Trinh CT (2012). Elucidating and reprogramming *Escherichia coli* metabolisms for obligate anaerobic n-butanol and isobutanol production. Appl Microbiol Biotechnol.

[8] Qi H, Li S, Zhao S, Huang D, Xia M, Wen J (2014). Model-driven redox pathway manipulation for improved isobutanol production in *Bacillus subtilis* complemented with experimental validation and metabolic profiling analysis. PLoS One.

[9] Foster JW, Moat AG (1980). Nicotinamide adenine dinucleotide biosynthesis and pyridine nucleotide cycle metabolism in microbial systems. Microbiol Rev.

[10] Lee WH, Kim JW, Park EH, Han NS, Kim MD, Seo JH (2013). Effects of NADH kinase on NADPH-dependent biotransformation processes in *Escherichia coli*. Appl Microbiol Biotechnol.

[11] King ZA, Feist AM (2014). Optimal cofactor swapping can increase the theoretical yield for chemical production in *Escherichia coli* and *Saccharomyces cerevisiae*. Metab Eng.

[12] Huang D, Li S, Xia M, Wen J, Jia X (2013). Genome-scale metabolic network guided engineering of *Streptomyces tsukubaensis* for FK506 production improvement. Microb Cell Fact.

[13] Shi A, Zhu X, Lu J, Zhang X, Ma Y (2013). Activating transhydrogenase and NAD kinase in combination for improving isobutanol production. Metab Eng.

[14] Bro C, Regenberg B, Förster J, Nielsen J (2006). In silico aided metabolic engineering of *Saccharomyces cerevisiae* for improved bioethanol production. Metab Eng.

[15] Singh A, Cher Soh K, Hatzimanikatis V, Gill RT (2011). Manipulating redox and ATP balancing for improved production of succinate in *Escherichia coli*. Metab Eng..

[16] Nocon J, Steiger MG, Pfeffer M, Sohn SB, Kim TY, Maurer M (2014). Model based engineering of *Pichia pastoris* central metabolism enhances recombinant protein production. Metab Eng.

[17] Bastian S, Liu X, Meyerowitz JT, Snow CD, Chen MM, Arnold FH (2011). Engineered ketol-acid reductoisomerase and alcohol dehydrogenase enable anaerobic 2-methylpropan-1-ol production at theoretical yield in *Escherichia coli*. Metab Eng.

[18] Bommareddy RR, Chen Z, Rappert S, Zeng AP (2014). A de novo NADPH generation pathway for improving lysine production of *Corynebacterium glutamicum* by rational design of the coenzyme specificity of glyceraldehyde 3-phosphate dehydrogenase. Metab Eng.

[19] Takeno S, Murata R, Kobayashi R, Mitsuhashi S, Ikeda M (2010). Engineering of *Corynebacterium glutamicum* with an NADPH-generating glycolytic pathway for l-lysine production. Appl Environ Microbiol.

[20] Alper H, Fischer C, Nevoigt E, Stephanopoulos G (2005). Tuning genetic control through promoter engineering. Proc Natl Acad Sci USA.

[21] Meynial-Salles I, Cervin MA, Soucaille P (2005). New tool for metabolic pathway engineering in *Escherichia coli*: one-step method to modulate expression of chromosomal genes. Appl Environ Microbiol.

[22] Ji XJ, Xia ZF, Fu NH, Nie ZK, Shen MQ, Tian QQ (2013). Cofactor engineering through heterologous expression of an NADH oxidase and its impact on metabolic flux redistribution in *Klebsiella pneumoniae*. Biotechnol Biofuels.

[23] Boghigian BA, Lee K, Pfeifer BA (2010). Computational analysis of phenotypic space in heterologous polyketide biosynthesis—applications to *Escherichia coli*, *Bacillus subtilis*, and *Saccharomyces cerevisiae*. J Theor Biol.

[24] Meng H, Lu Z, Wang Y, Wang X, Zhang S (2011). In silico improvement of heterologous biosynthesis of erythromycin precursor 6-deoxyerythronolide B in *Escherichia coli*. Biotechnol Bioprocess Eng.

[25] Csonka LN, Fraenkel DG (1977). Pathways of NADPH formation in *Escherichia coli*. J Biol Chem.

[26] Sauer U, Canonaco F, Heri S, Perrenoud A, Fischer E (2004). The soluble and membrane-bound transhydrogenases UdhA and PntAB have divergent functions in NADPH metabolism of *Escherichia coli*. J Biol Chem.

[27] Fuhrer T, Sauer U (2009). Different biochemical mechanisms ensure network-wide balancing of reducing equivalents in microbial metabolism. J Bacteriol.

[28] Centeno-Leija S, Utrilla J, Flores N, Rodriguez A, Gosset G, Martinez A (2013). Metabolic and transcriptional response of *Escherichia coli* with a NADP(+)-dependent glyceraldehyde 3-phosphate dehydrogenase from *Streptococcus mutans*. Antonie Van Leeuwenhoek.

[29] Zhu J, Shimizu K (2004). The effect of pfl gene knockout on the metabolism for optically pure d-lactate production by *Escherichia coli*. Appl Microbiol Biotechnol.

[30] Singh A, Lynch MD, Gill RT (2009). Genes restoring redox balance in fermentation-deficient *E. coli* NZN111. Metab Eng.

[31] Zhou L, Zuo ZR, Chen XZ, Niu DD, Tian KM, Prior BA (2011). Evaluation of genetic manipulation strategies on d-lactate production by Escherichia coli. Curr Microbiol.

[32] Canonaco F, Hess TA, Heri S, Wang T, Szyperski T, Sauer U (2001). Metabolic flux response to phosphoglucose isomerase knock-out in Escherichia coli and impact of overexpression of the soluble transhydrogenase UdhA. FEMS Microbiol Lett.

[33] Martínez I, Zhu J, Lin H, Bennett GN, San KY (2008). Replacing *Escherichia coli* NAD-dependent glyceraldehyde 3-phosphate dehydrogenase (GAPDH) with a NADP-dependent enzyme from *Clostridium acetobutylicum* facilitates NADPH dependent pathways. Metab Eng.

[34] Iddar A, Valverde F, Serrano A, Soukri A (2002). Expression, purification, and characterization of recombinant nonphosphorylating NADP-dependent glyceraldehyde-3-phosphate dehydrogenase from *Clostridium acetobutylicum*. Protein Expr Purif.

[35] Atsumi S, Wu TY, Eckl EM, Hawkins SD, Buelter T, Liao JC (2010). Engineering the isobutanol biosynthetic pathway in *Escherichia coli* by comparison of three aldehyde reductase/alcohol dehydrogenase genes. Appl Microbiol Biotechnol.

[36] Zhou YJ, Yang W, Wang L, Zhu Z, Zhang S, Zhao ZK (2013). Engineering NAD+ availability for *Escherichia coli* whole-cell biocatalysis: a case study for dihydroxyacetone production. Microb Cell Fact.

[37] Wimpenny JW, Firth A (1972). Levels of nicotinamide adenine dinucleotide and reduced nicotinamide adenine dinucleotide in facultative bacteria and the effect of oxygen. J Bacteriol.

[38] Jensen PR, Michelsen O (1992). Carbon and energy metabolism of atp mutants of *Escherichia coli*. J Bacteriol.

[39] Naseem R, Wann KT, Holland IB, Campbell AK (2009). ATP regulates calcium efflux and growth in *E. coli*. J Mol Biol.

[40] Ying W (2008). NAD^+^/NADH and NADP^+^/NADPH in cellular functions and cell death: regulation and biological consequences. Antioxid Redox Signal.

[41] Brynildsen MP, Liao JC (2009). An integrated network approach identifies the isobutanol response network of *Escherichia coli*. Mol Syst Biol.

[42] Kim I, Yun H, Jin I (2007). Comparative proteomic analyses of the yeast *Saccharomyces cerevisiae* KNU5377 strain against menadione-induced oxidative stress. J Microbiol Biotechnol.

[43] Schreiber W, Dürre P (1999). The glyceraldehyde-3-phosphate dehydrogenase of *Clostridium acetobutylicum*: isolation and purification of the enzyme, and sequencing and localization of the gap gene within a cluster of other glycolytic genes. Microbiology.

[44] Lim JH, Seo SW, Kim SY, Jung GY (2013). Model-driven rebalancing of the intracellular redox state for optimization of a heterologous *n*-butanol pathway in *Escherichia coli*. Metab Eng.

[45] Schmittgen TD, Livak KJ (2008). Analyzing real-time PCR data by the comparative C(T) method. Nat Protoc.

[46] Ma J, Gou D, Liang L, Liu R, Chen X, Zhang C (2013). Enhancement of succinate production by metabolically engineered Escherichia coli with co-expression of nicotinic acid phosphoribosyltransferase and pyruvate carboxylase. Appl Microbiol Biotechnol.

[47] Kandasamy V, Vaidyanathan H, Djurdjevic I, Jayamani E, Ramachandran KB, Buckel W (2013). Engineering Escherichia coli with acrylate pathway genes for propionic acid synthesis and its impact on mixed-acid fermentation. Appl Microbiol Biotechnol.

[48] Zhu LW, Li XH, Zhang L, Li HM, Liu JH, Yuan ZP (2013). Activation of glyoxylate pathway without the activation of its related gene in succinate-producing engineered *Escherichia coli*. Metab Eng.

[49] Sambrook J, Russell DW (2001). Molecular cloning: a laboratory manual.

[50] Orth JD, Conrad TM, Na J, Lerman JA, Nam H, Feist AM (2011). A comprehensive genome-scale reconstruction of *Escherichia coli* metabolism—2011. Mol Syst Biol.

[51] Johansson T, Oswald C, Pedersen A, Törnroth S, Okvist M, Karlsson BG (2005). X-ray structure of domain I of the proton-pumping membrane protein transhydrogenase from *Escherichia coli*. J Mol Biol.

[52] Orth JD (2012) Systems biology analysis of *Escherichia coli* for discovery and metabolic engineering. Ph.D. thesis, University of California, San Diego

[53] Lin Z, Xu Z, Li Y, Wang Z, Chen T, Zhao X (2012). Metabolic engineering of *Escherichia coli* for the production of riboflavin. Microb Cell Fact.

[54] Fu J, Wang Z, Chen T, Liu W, Shi T, Wang G (2014). NADH plays the vital role for chiral pure d-(-)-2,3-butanediol production in *Bacillus subtilis* under limited oxygen conditions. Biotechnol Bioeng.

[55] Li S, Xu N, Liu L, Chen J (2013). Engineering of carboligase activity reaction in *Candida glabrata* for acetoin production. Metab Eng.

