# Supplementary material for: Model-driven intracellular redox status modulation for increasing isobutanol production in Escherichia coli
Source: Biotechnol Biofuels. 2015 Aug 1;8:108. doi: 10.1186/s13068-015-0291-2 (PMC4522091; doi:10.1186/s13068-015-0291-2)
Supplement: Additional file 1: — Supplementary supporting data. [file 13068_2015_291_MOESM1_ESM.docx]

**Additional file 1**

**Table S1**. Primers used in this study

| **Primer** | **Sequence** |
| --- | --- |
| alsS-F | 5’-GCGCGGATCCAGGAGATATACCATGTTGACAAAAGCAACAAAAGAAC-3’ |
| alsS-R | 5’-ACAGTCGACACAAGCACGCGTCTAGAGAGCTTTCGTTTTCATGAGTT-3’ |
| ilvC-F | 5’-AGCGACGCGTTCACGAGGAATCACCATGGCTAACTAC-3’ |
| ilvC-R | 5’-TACGGTCGACTAATCGAGATCTTTAACCCGCAACAGCAATACGTTTC-3’ |
| ilvD-F | 5’-CAGAAGATCTAGGAGATATACCATGCCTAAGTACCGTTCCGCCACCACCACT-3’ |
| ilvD-R | 5’-TGCCGTCGACTTAACCCCCCAGTTTCGATTTATC-3’ |
| kivd-F | 5’-GCTGGATCCAGGAGGGTATAGCTATGTATACAGTAGGAGATTACCTAT-3’ |
| kivd-R | 5’-GCCTCTAGAGCCATGGTTATGATTTATTTTGTTCAGCAAAT-3’ |
| yqhD-F | 5’-TGCTCTAGAAGGAGATATACCATGAACAACTTTAATCTGCACACCCCAAC-3’ |
| yqhD-R | 5’-AAAACTGCAGACGAAATGCCCGAAAACGAA-3’ |
| PgapA-F | 5’-CGGGGTACCTTGCTCACATCTCACTTTAATCG-3’ |
| PgapA-R | 5’-TCGCGTCGACCTAGGCACGCGTATATTCCACCAGCTATTTGTTAGTG-3’ |
| PLO-F | 5’-CACGGTCGACGTAACTGGTACCCCAAGTTTACTCATATATACTTTAG-3’ |
| PLO-R | 5’-ACTCCTGCAGATCCCCGGATCCAGAGCTCGAATTCTGTGT-3’ |
| TB-F | 5’-CAGGCTGCAGAGCTTGGCTGTTTTGGC-3’ |
| TB-R | 5’-AAACGTCGACAGAGCACTCGAGGTCTGACAGTTACCAAT-3’ |
| OC-F | 5’-CGGGAGATCTGAACACCTACATCTGTATTAAC-3’ |
| OC-R | 5’-CGGTACGCGTTTTAACTGTGATAAACTAC-3’ |
| PT-F | 5’-TGGAACGCGTGCTTATCATCGACTGCACGG-3’ |
| PT-R | 5’-GGCTAGATCTCGGATACATATTTGAATGTATT-3’ |
| gapN-F | 5’-GGCACGCGTAGGAGATATACCATGTTTGAAAATATATCATC-3’ |
| gapN-R | 5’-AGCGGTCGACGTGAGATTGGATAGAATAAAATTATAGGTT-3’ |
| gapC-F | 5’-ACGGACGCGTGCATGAGGTAGTTAGAATGGC-3 |
| gapC-R | 5-TCCCCGTCGACGCAAATTAATTAATGAGCGC-3 |
| BBa_J23105-F | 5’-ACGGGGTACCTTTACGGCTAGCTCAGTCCTAGGTACTATGCTAGCTATACGCGTAGGAGATATACCATG-3’ |
| BBa_J23106-F | 5’-ACGGGGTACCtttacggctagctcagtcctaggtatagtgctagcTATACGCGTAGGAGATATACCATG-3’ |
| BBa_J23118-F | 5’-ACGGGGTACCTTGACGGCTAGCTCAGTCCTAGGTATTGTGCTAGCTATACGCGTAGGAGATATACCATG-3’ |
| BBa_J23102-F | 5’-ACGGGGTACCttgacagctagctcagtcctaggtactgtgctagcTATACGCGTAGGAGATATACCATG-3’ |
| BBa_J23100-F | 5’-ACGGGGTACCTTGACGGCTAGCTCAGTCCTAGGTACAGTGCTAGCTATACGCGTAGGAGATATACCATG-3’ |
| **Primers for determining *gapN* and 16SrRNA genes expression level by RT-PCR** | |
| 16SrRNA-rtF | 5’- AGTTAATACCTTTGCTCATTG-3’ |
| 16SrRNA-rtR | 5’-GCTTGCCAGTATCAGATG-3’ |
| gapN-rtF | 5’-GCCCAGATCACTTCCCATTCC-3’ |
| gapN-rtR | 5’-TGCCTTGTCATTGCCTCTATACTG-3’ |
| gapC-rtF | 5’-ACTACAATTCATGCGTTCACT-3’ |
| gapC-rtR | 5’-CATAGCAGCGTTGATTTCTT-3’ |
| alsS-rtF | 5’-ACGGCGGTTTCTTATTC-3’ |
| alsS-rtR | 5’-GGACCTTCAGCGTTCAT-3’ |
| IlvC-rtF | 5’-ACGAAGAACTGATCCCACAGGC-3’ |
| IlvC-rtR | 5’-TTCCGCAACGAAGGACGATT-3’ |
| IlvD-rtF | 5’-TCCGATCAGATCATCAAGC-3’ |
| IlvD-rtR | 5’-TTCAACAATGCGTTTACCAG-3’ |
| kivd-rtF | 5’-CAGCCGACTTCATCC-3’ |
| kivd-rtR | 5’-CCATAGGCGGTCTTG-3’ |
| yqhD-rtF | 5’-CCCGCATTCTGTTTGG-3’ |
| yqhD -rtR | 5’-CCTGCTTGTCGCCTGT-3’ |

**Table S2**. The transhydrogenase reaction encoded by *pntAB* used in the model of this work and iJO1366 model

| **Model** | **Reaction** | **Reference** |
| --- | --- | --- |
| iJO1366 | nadh[c] + nadp[c] + 2 h[p] -> 2 h[c] + nad[c] + nadph[c] | a |
| Model used in this work | nadh[c] + nadp[c] + h[p] -> h[c] + nad[c] + nadph[c] | b |

a: Orth JD, Conrad TM, Na J, Lerman JA, Nam H, Feist AM, Palsson BØ. A comprehensive genome-scale reconstruction of *Escherichia coli* metabolism--2011. Mol. Syst. Biol. 2011; 7:535.

b: Johansson T, Oswald C, Pedersen A, Törnroth S, Okvist M, Karlsson BG, Rydström J, Krengel U. X-ray structure of domain I of the proton-pumping membrane protein transhydrogenase from *Escherichia coli*. J. Mol. Biol. 2005; 352 (2):299-312.

**Table S3**. Isobutanol biosynthesis and transport reactions added into the metabolic network model of strain LA02

| **Reaction name** | **Reaction** | **Gene** |
| --- | --- | --- |
| alpha-ketoisovalerate decarboxylase | 3mob[c] + h[c] --> 2mpal[c] + co2[c] | *Kivd* |
| alcohol dehydrogenase (NADP^+^) | 2mpal[c] + nadph[c] + h[c] --> 2mpol[c] + nadp[c] | *yqhd* |
| isobutanol exchange | 2mpol[e] <==> |  |
| isobutanol transport via diffusion (extracellular to periplasm) | 2mpol[e] <==> 2mpol[p] |  |
| isobutanol transport via diffusion (intracellular to periplasm) | 2mpol[p] <==> 2mpol[c] |  |

3mob: 3-Methyl-2-oxobutanoate, 2mpal: 2-Methlpropanal, 2mpol: isobutanol

**Table S4**. The candidate reactions for targets prediction of redox status rebalance

| **Reaction abbreviations** | **Reaction name** | **gene** |
| --- | --- | --- |
| **NADPH metabolism** | | |
| GLUDy**^a^** | glutamate dehydrogenase (NADP) | *gdhA* |
| FLDR2**^a^** | flavodoxin reductase (NADPH) | *fldA&Fpr or fldB&Fpr* |
| G5SD**^a^** | glutamate-5-semialdehyde dehydrogenase | *proA* |
| GTHOr**^a^** | glutathione oxidoreductase | *gapFd* |
| MTHFD**^b^** | methylenetetrahydrofolate dehydrogenase (NADP) | *folD* |
| G6PDH2r**^b^** | glucose 6-phosphate dehydrogenase | *zwf* |
| GND**^b^** | phosphogluconate dehydrogenase | *gnd* |
| ICDHyr**^b^** | isocitrate dehydrogenase (NADP) | *icd* |
| THD2pp**^a^** | NAD(P) transhydrogenase (periplasm) | *pnt* |
| **NADH metabolism** | | |
| ACALD**^b^** | acetaldehyde dehydrogenase (acetylating) | *mhpF or adhE* |
| ALCD2x**^b^** | alcohol dehydrogenase (ethanol) | *adhP or adhE or frmA* |
| NADH16pp**^b^** | NADH dehydrogenase (periplasm) | *nuo* |
| LDH_D**^b^** | D-lactate dehydrogenase | *ldh* |
| FADRx**^b^** | FAD reductase | *fre* |
| DMPPS**^a^** | 1-hydroxy-2-methyl-2-(E)-butenyl 4-diphosphate reductase (dmpp) | *lytB* |
| IMPD**^a^** | IMP dehydrogenase | *guaB* |
| MDH**^b^** | malate dehydrogenase | *mdh* |
| PGCD**^b^** | phosphoglycerate dehydrogenase | *serA* |
| PDH**^b^** | pyruvate dehydrogenase | *aceEec&aceFec&lpdA* |
| GAPD**^b^** | glyceraldehyde-3-phosphate dehydrogenase | *gapA* |
| GLYCL**^a^** | Glycine Cleavage System | *gcvH&gcvP&gcvT&lpdA* |
| THRD^a^ | L-threonine dehydrogenase | *tdh* |
| TRSARr**^a^** | tartronate semialdehyde reductase | *glxR or garR* |

**^a^** These 10 potential targets were different with the targets reported by King and Feist (2014)

**^b^** These 13 potential targets were also reported by King and Feist (2014)

King ZA, Feist AM. Optimal cofactor swapping can increase the theoretical yield for chemical production in Escherichia coli and Saccharomyces cerevisiae. Metab Eng. 2014; 24:117-128.

**Table S5.** GAPDH activity assays of cell extracts of the *gapC-*expressing strain LA03 and *gapN*-expressing strain LA04

| Strain | NAD^+^-dependent GAPDH activity  (IU/mg protein) | NADP^+^-dependent GAPDH activity  (IU/mg protein) |
| --- | --- | --- |
| *E.coli* LA02 | 0.571±0.04 | ＜0.02 |
| *E.coli* LA03 | 0.785±0.05 | 0.096±0.02 |
| *E.coli* LA04 | 0.608±0.03 | 0.425±0.05 |

**Table S6** Prediction results of the potential redox targets with *f_PH_* no more than 1.

| Reaction name | Reaction stoichiometry | Strategy | Special  growth rate (h^-1^) | ISB special product rate (mM/g/h) | *f_PH_* |
| --- | --- | --- | --- | --- | --- |
| GND | 6-Phospho-D-gluconate +NADP^+^→ CO_2_ + NADPH + D-Ribulose 5-phosphate | Over express | 0.0914 | 0.645 | 0.953 |
| MTHFD | 5,10-Methylenetetrahydrofolate + NADP^+^ ↔ NADPH + 5,10-Methenyltetrahydrofolate | Over express | 0.0929 | 0.646 | 0.965 |
| ICDHyr | Isocitrate + NADP^+^ ↔ NADPH + 2-Oxoglutarate + CO_2_ | Over express | 0.0925 | 0.633 | 0.921 |
| NADH16pp | 4 H^+^(extracellular) + NADH + Ubiquinone-8 →NAD^+^ + Ubiquinol-8 + 3 H^+^( intracellular) | knock out | 0.0951 | 0.650 | 1.000 |
| FADRx | fad + H^+^(extracellular) + NADH → fadh2 + NAD^+^ | knock out | 0.0944 | 0.641 | 0.966 |
| DMPPS | 1-hydroxy-2-methyl-2-(E)-butenyl 4-diphosphate + H^+^ + NADH → Dimethylallyl diphosphate + H_2_O + NAD^+^ | knock out | 0.0951 | 0.650 | 1.000 |
| MDH | L-Malate + NAD^+^↔ H^+^ + NADH + Oxaloacetate | knock out | 0.0942 | 0.645 | 0.975 |
| PDH | Coenzyme A + NAD^+^ + Pyruvate →Acetyl-CoA + CO_2_ + NADH | cofactor swap | 0.0941 | 0.649 | 0.986 |
| IMPD | H_2_O + IMP + NAD^+^ →H^+^ + NADH + Xanthosine 5'-phosphate | cofactor swap | 0.0951 | 0.650 | 1.000 |
| GLYCL | Glycine + NAD^+^ + 5,6,7,8-Tetrahydrofolate → CO_2_ + 5,10-Methylenetetrahydrofolate + NADH + NH_4_ | cofactor swap | 0.0951 | 0.650 | 1.000 |
| THRD | NAD^+^ + L-Threonine → L-2-Amino-3-oxobutanoate + H^+^ + NADH | cofactor swap | 0.0951 | 0.650 | 1.000 |
| TRSARr | 2-Hydroxy-3-oxopropanoate + H^+^ + NADH ↔ (R)-Glycerate + NAD^+^ | cofactor swap | 0.0951 | 0.650 | 1.000 |

fad: Flavin adenine dinucleotide oxidized, fadh2: Flavin adenine dinucleotide reduced

**

**

**Figure S1 Metabolism of NADH and NADPH in strain LA02 simulated by FBA with the experiment-determining fermenter parameters of strain LA02 as constraints, and strategies of redox status improvement used in modeling.** The blue arrows indicated the NADH metabolism; the red arrows indicated the NADPH metabolism. The total fluxs of NADH and NADPH were respectively 16.20 and 3.44 mmol/g/h, and flux ratios of some reactions involving in NADH and NADPH metabolism were given as percentage. To improve intercellular redox status (increasing NADPH availability and decreasing NADH level) for isobutanol biosynthesis, ①the NADPH-generating reactions could be overexpression, ②the NADH or NADPH-consuming reactions could be knocked out, ③the NADH-generating or NADPH-consuming reactions could be cofactor swapped.





**Figure S2. Comparison of intracellular redox cofactors in stationary phase for strain LA02, LA03 and LA04.**
