# Supplementary material for: Model-driven intracellular redox status modulation for increasing isobutanol production in Escherichia coli
Source: Biotechnol Biofuels. 2015 Aug 1;8:108. doi: 10.1186/s13068-015-0291-2 (PMC4522091; doi:10.1186/s13068-015-0291-2)
Supplement: Additional file 2: — Construction procedures of pTRCLA and pACYCLA. [file 13068_2015_291_MOESM2_ESM.docx]

**Additional file 2**

**Construction procedures of pTRCLA and pACYCLA**

A fragment (PLO) containing *trc* promoter, *LaIq* operator, and pMB1 (pBR322) origin of replication (*ori*) was PCR amplified using a pair of primers (PLO-F and PLO-R) from pTRC99a. Another fragment (TB) containing *rrnB* terminator and ampicillin resistance gene was PCR amplified using a pair of primers (TB-F and TB-R) from pTRC99a. These two fragments were digested with *Kpn*I and *Pst*I, and then ligated by T4 DNA ligase, yielding pTRCLA.

A fragment (PT) containing *trc* promoter, multiple cloning site sequences and *rrnB* terminator was PCR amplified using a pair of primers (PT-F and PT-R) from pTRC99a. Another fragment (OC) containing p15A *ori* and chloramphenicol resistance gene was PCR amplified using a pair of primers (OC-F and OC-R) from pACYC184. These two fragments were digested with *Bgl*II and *Mlu*I, and then ligated by T4 DNA ligase, creating pACYCLA.

Primers were given in Additional file 1: Table S1.
