# Supplementary material for: Model-driven intracellular redox status modulation for increasing isobutanol production in Escherichia coli
Source: Biotechnol Biofuels. 2015 Aug 1;8:108. doi: 10.1186/s13068-015-0291-2 (PMC4522091; doi:10.1186/s13068-015-0291-2)
Supplement: Additional file 3: — Model verification. [file 13068_2015_291_MOESM3_ESM.docx]

**Additional file 3**

**Model verification**

According to Huang et al. (2013), the model was verified with the constraints using the parameters of strain LA02 fermentation determined experimentally. The details were as follows, the lower bound for isobutanol transport flux was set as 0.65 mmol/g/h, the lower bound of glucose uptake rate was set as -7 mmol/g/h, the lower bound of lactate, ethanol, acetate and succinate was respectively set as 9.45, 1.05, 0.70 and 0.21 mmol/g/h, the lower bound of all amino acids uptake rate was set as -0.05 mmol/g/h according to Huang et al. (2013), and the lower bound of oxygen uptake rate was set to be -2 mmol/g/h according to Ng et al. (2012). Then, the special growth rate was simulated to be 0.095 using FBA algorithm. Finally, the predicted special growth rate was very close to the experimental observed special growth rate (0.105), and the error of 9.5% was within the acceptable range of 10% according to Selvarasu, et al. (2009)

References:

Huang D., Li S., Xia M., Wen J., Jia X., 2013. Genome-scale metabolic network guided engineering of *Streptomyces tsukubaensis* for FK506 production improvement. Microb. Cell Fact. 12, 52.

Ng C.Y., Jung M.Y., Lee J., Oh M.K., 2012. Production of 2,3-butanediol in *Saccharomyces cerevisiae* by in silico aided metabolic engineering. Microb. Cell Fact. 11, 68.

Selvarasu S, Ow D.S., Lee S.Y., Lee M.M., Oh S.K., Karimi I.A., Lee D.Y. 2009. Characterizing *Escherichia coli* DH5alpha growth and metabolism in a complex medium using genome-scale flux analysis. Biotechnol. Bioeng. 102(3), 923-934.
